# Partnership for Health-2, A Web-Based Versus Print Smoking Cessation Intervention for Childhood and Young Adult Cancer Survivors: Randomized Comparative Effectiveness Study

**DOI:** 10.2196/jmir.2533

**Published:** 2013-11-05

**Authors:** Karen M Emmons, Elaine Puleo, Kim Sprunck-Harrild, Jennifer Ford, Jamie S Ostroff, David Hodgson, Mark Greenberg, Lisa Diller, Janet de Moor, Vida Tyc

**Affiliations:** ^1^Dana-Farber Cancer InstituteBoston, MAUnited States; ^2^Harvard School of Public HealthBoston, MAUnited States; ^3^University of MassachusettsAmherst, MAUnited States; ^4^Memorial Sloan Kettering Cancer CenterNew York, NYUnited States; ^5^Princess Margaret HospitalToronto, ONCanada; ^6^Hospital for Sick ChildrenToronto, ONCanada; ^7^Harvard Medical SchoolBoston, MAUnited States; ^8^Ohio State University College of Public HealthColumbus, OHUnited States; ^9^St. Judes Children's Research HospitalMemphis, TNUnited States

**Keywords:** cancer survivors, smoking cessation, cancer prevention

## Abstract

**Background:**

Smoking among cancer survivors increases the risk of late effects and second cancers. This article reports on Partnership for Health-2 (PFH-2)—an effort to develop an effective and scalable version of Partnership for Health (PFH), which was a previously tested peer-delivered telephone counseling program that doubled smoking cessation rates among childhood cancer survivors who smoke.

**Objective:**

This paper presents results from a randomized controlled trial evaluating the effectiveness of PFH-2 in targeted and tailored Web-based versus print formats. The overall goal was to determine whether the intervention outcomes in these self-guided scalable formats approximate what was found in a more intensive telephone counseling program.

**Methods:**

This study was a randomized controlled trial with a 15-month follow-up that included 374 smokers who were survivors of childhood or young adult cancers, recruited from five survivorship clinics. Participants were randomly assigned to a Web-based or print format of the PFH intervention; all had access to free pharmacotherapy. The website was designed to provide new content at each log-on, and a peer counselor moderated a forum/chat feature. The primary outcome was smoking status at 15 months post randomization.

**Results:**

In total, 58.3% (77/132) of Web participants logged on at least once (mean visits 3.25). Using multiple imputation methods for missing data, there were similar rates of cessation in the two arms (print: 20/128, 15.6%; Web: 33/201, 6.4%), and no differences in quit attempts or readiness to quit. The quit rates were equivalent to those found in our previous telephone counseling intervention. There were high rates of satisfaction with both of the PFH-2 interventions.

**Conclusions:**

The print and Web formats yielded equivalent levels of success to those found with our telephone-delivered intervention and are comparable to other Internet treatment studies. This study provides important options for survivorship programs that may not have resources for interpersonal forms of cessation counseling. Efforts to increase patient use of the interventions may result in higher cessation rates.

**Trial Registration:**

Clinicaltrials.gov NCT00588107; http://clinicaltrials.gov/ct2/show/NCT00588107 (Archived by WebCite at http://www.webcitation.org/6K1gJtFEC).

## Introduction

Remarkable improvements in the treatment and long-term survival of childhood and young adult cancer survivors have resulted in prevention of adverse late effects and second primary cancers being a key part of survivorship care [1-5]. Smoking rates among this population are substantial [6-8]. In the Childhood Cancer Survivor Study, the largest US cohort of childhood cancer survivors, 28% of survivors reported having smoked at least 100 cigarettes in their lifetime and 17% reported current cigarette smoking [6]. In the British Childhood Cancer Survivor Study, a population-based cohort of 17,981 survivors, 20.0% were current regular smokers and 29.8% were regular smokers but no longer are [8].

This team’s previous work has demonstrated the efficacy of Partnership for Health (PFH)*,* a survivor-focused peer-delivered telephone counseling intervention for smoking cessation. PFH led to a doubling in quit rates compared with usual care and the intervention effect was sustained over 2-5 years of follow-up [9,10]. The connection that the peer-delivered intervention provided between survivors was an important way to engage participants in the intervention. However, it is challenging to scale interventions that include ongoing counseling.

A recent evaluation of existing infrastructure for delivering smoking cessation services in the context of survivorship programs revealed relatively few resources; only 3% of programs assessed smoking status at every visit, as recommended by Public Health Service (PHS) guidelines, and only 25% offered cessation services [11]. Further, cancer survivors are quite geographically dispersed and thus, effective interventions are needed that can be scaled easily and delivered remotely regardless of survivors’ location. The present study focused on the adaptation of the PFH peer-delivered intervention for a Web- and print-based format as a way to increase the intervention’s dissemination potential and sustainability. We selected Web- and print-based interventions because of the relatively high penetration of Internet access in the target age group [12] and because these formats could be integrated easily into standard practice at survivorship programs across the country, compared with telephone-based interventions.

This paper presents results from a randomized control trial evaluating the effectiveness of Partnership for Health-2 (PFH-2) in targeted and tailored Web-based versus print formats. The overall goal was to determine the outcomes for these self-guided scalable formats and whether they approximate what was found in a more intensive telephone counseling program. Our hypothesis was that the Web format would yield higher quit rates than print and would be similar in effectiveness to that found in the original PFH counseling intervention.

## Methods

### Setting

PFH-2 was conducted in collaboration with five cancer centers in the United States and Canada, with Institutional Review Board (IRB) approval at all sites. The study was also advertised on childhood and young adult survivorship websites. Eligibility included: diagnosed with cancer before age 35, currently between ages 18-55, completed cancer treatment for ≥2 years, mentally able to provide informed consent, reachable by telephone, able to speak English, and a current smoker (defined as smoking within the previous 30 days). Participants were informed that the study was examining different ways to deliver health information, including information about tobacco use, to survivors. They were not required to be interested in smoking cessation in order to participate. Baseline data collection began on December 2005 and follow-up data collection ended in October 2009.

A preliminary screen for eligiblity was performed at each site via medical record review or brief telephone screening. After consent for sharing contact information was obtained, contact information was forwarded to the survey team to verify eligibility, obtain informed consent for study participation, and administer the baseline telephone survey.

### PFH-2 Study Design

PFH-2 was a stratified randomized controlled trial with cancer center as strata. The goal was to test two scalable intervention formats for smoking cessation among childhood and young adult survivors, developed from an evidence-based intervention, and to determine whether the Web intervention, with the advantages that an interactive website has to offer, would outperform tailored and targeted print materials. Participants were randomized to one of two intervention conditions within strata, in a 5:3 randomization scheme: (1) PFH-2 Print Materials Intervention, or (2) PFH-2 Web Intervention. The random allocation sequence was generated by the study biostatistician. Randomization was done by the survey team and supervised by the biostatistician, following completion of the baseline survey. Study design is provided in detail elsewhere [13]. Intervention goals for both conditions included: (1) assess and enhance motivation to change, (2) address ambivalence about behavior change, (3) provide social support, (4) assess and build self-efficacy, (5) increase awareness of risks, (6) identify and address barriers to change, and (7) address nicotine dependence. Both conditions included: (1) a letter encouraging smoking cessation from the site oncologist, developed based on the principles of the National Cancer Institute’s “5 A’s” smoking counseling guidelines, (2) free pharmacotherapy for participants and spouses/significant others who want to quit, and (3) tailored and targeted self-help content (Web or print) addressing participant-specific barriers to change and other survivor-related topics of interest. The intervention period was 6 months and a follow-up survey was completed by telephone at 15 months after randomization.

### PFH-2 Print Materials Intervention

The Print Materials arm received tailored and targeted self-help materials that were developed for the peer counselor condition in PFH-1. The materials were organized into a series of manuals, based on readiness to change [14], that addressed participant-specific barriers to change and other survivor-related topics of interest (eg, addressing depression, handling stress, managing weight). The manuals were designed to be as interactive as possible, with worksheets and opportunities for personalizing the content included throughout. Testimonials and stories of other survivors’ experiences were used to provide the survivor-to-survivor connection. Both PFH-2 conditions included other key features of the original PFH peer-delivered intervention, including a letter encouraging smoking cessation from the site oncologist and free pharmacotherapy (nicotine patch or Zyban) for themselves and any smoking partner/spouse who wished to quit. Those who were interested in pharmacotherapy contacted study staff and provided permission to contact their health care provider for approval using a fax-back form. We also sent the provider a copy of the pamphlet “Helping Smokers Quit: A guide for primary care clinicians” (US Department of Health and Human Service/Agency for Health Care Policy and Research) and a basic fact sheet about adult survivors of cancer. The pharmacotherapy was also sent to the provider and distributed to the patient by his or her office.

**Figure 1:**
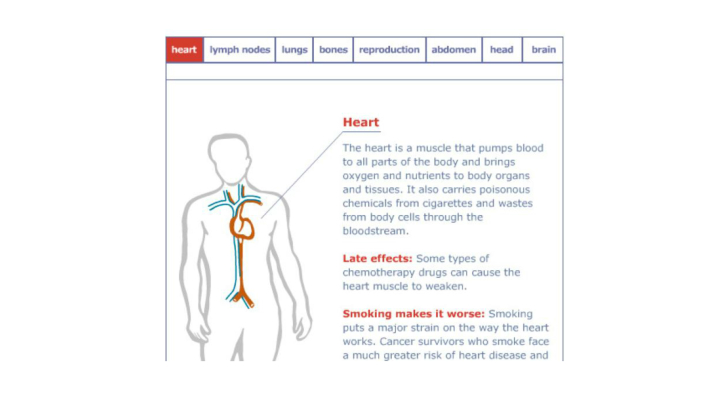
Illustration of the interaction of smoking and cancer treatment/late effects.

A baseline feedback report (BFR) was generated for each individual and sent between 5 and 10 business days following completion of the baseline survey. The BFR reflected information that patients gave on the baseline survey about their readiness to quit, perceived risk, nicotine dependence, and, based on these factors, which intervention manual to start with, drawing on approaches we have used previously [11,15]. The BFR also gave basic facts about cancer treatment and illustrated how cancer, cancer treatment, and smoking affects many of the same organs (see Figure 1). The Print Materials condition was designed to reflect as many of the realities as possible of how the intervention might be utilized when scaled to existing cancer survivorship clinics.

### PFH-2 Web Intervention

Within 5-10 business days following completion of the baseline survey, those assigned to the Web intervention were sent log-on information. The Web intervention consisted of seven discrete tailored sessions designed to parallel the counseling sessions of the original PFH study and mirror the basic content of the PFH-2 print materials. The content was dynamically tailored, matching the participants’ stage of readiness. Upon first log-on, participants saw their BFR, described above, on their home page. The home page also highlighted active components of the intervention that participants had not yet navigated. Patients saw refreshed content on the home page as they progressed from session to session. A peer counselor moderated the website’s discussion forum and served as a resource for questions.

Participants who never logged in or stopped using the site received additional email prompts that highlighted content that survivors might find particularly important or engaging, along with biweekly emailed newsletters. Those who had not accessed the website within 11 weeks were sent a final letter along with the print materials to increase the likelihood of some exposure to the intervention content and to approximate likely approaches in a clinic setting. Participants had access to the website for 6 months regardless of their log-in status. For quality assurance purposes, several “test participants” were created and followed throughout the implementation period to identify glitches or issues with the Web system. These “participants” were not included in the tracking data and did not impact on intervention delivery in any way.

### Measures

#### Sociodemographics and Medical History

Age, gender, race, ethnicity, marital status, education, height, weight, employment status, type of cancer, and cancer treatment were assessed.

#### Internet Access and Utilization

Internet access and utilization were measured with questions about whether participants owned a computer, whether they had access to the Internet at home and/or work, and how frequently they used a computer, email, and the Internet [16].

#### Smoking Behavior

“Smoking status” was assessed by self-reported assessment of smoking, even a puff, in the past 30 days. The bogus pipeline procedure was used to increase the accuracy of self-report, following standard implementation methods [17,18]. Specifically, participants were informed that they may be asked to provide a saliva sample to confirm their smoking status. This procedure has been found to improve the quality of self-reported smoking behavior. “Nicotine dependence” was assessed based on number of minutes after waking that participants smoked their first cigarette [19] (<30 minutes, nicotine dependent; ≥30 minutes, not nicotine dependent). “Quit attempts” were assessed by the number of quits in the previous 12 months with at least 24 hours abstinence. “Use of pharmacotherapy” was assessed with two questions about whether participants had ever used Zyban or nicotine replacement therapy to quit smoking.

#### Motivational Variables

“The Stages of Change Scale” assessed motivation to quit smoking [14], according to four categories: (1) precontemplation: not seriously thinking about quitting in the next 6 months; (2) contemplation: seriously thinking about quitting in the next 6 months; (3) preparation: intending to quit in the next month and have tried to quit in the past year; and (4) action: not currently smoking and quit within the past 6 months. “Self-efficacy” was assessed related to participants’ level of confidence that they could quit smoking in the next 1 and 6 months [7].

#### Household and Workplace Smoking Policy

Participants were asked about rules regarding smoking at home and work (smoking unrestricted inside the building/house, limited to certain rooms, or forbidden inside the building/house).

#### Psychological Variables

“Cancer-related distress” was assessed with the Intrusive Thoughts subscale of the Impact of Events Scale (IES), which measures frequency with which thoughts regarding cancer recurrence intrude into consciousness [20]. “Perceived control” was assessed with a 3-item scale that measured the degree to which participants felt they could control physical side effects, future health, and chance of a cancer recurrence [21]. “Perceived vulnerability” was assessed with a question about the likelihood of experiencing serious health problems in the future [11,22]. “Depression” was measured with the 2-item Prime MD scale [23].

#### Contact With the Health Care System

Participants were asked if they had a regular health care provider and if they had been seen by their primary care physician or their oncologist in the past year.

#### Intervention Use

Use of the Web-based intervention was assessed using Web analytics. Use of the print intervention was assessed on the follow-up survey, with questions about the percentage of materials read and frequency of use.

### Data Analysis

Original sample size calculations were adjusted due to the discovery during implementation that the participating survivorship programs’ estimates of smokers were an over-estimate. Thus, we used a 5:3 allocation scheme to ensure an adequate sample size to evaluate the use of the Web-based intervention. With this approach, we had 71% power to detect a difference of 9% in quit rate between the Web and print groups. Baseline comparisons of patient characteristics between intervention arms were assessed. Depression level was the only variable of significance in this comparison and was, in addition to site, controlled for in all future analyses. All outcome analyses were conducted using multiple imputation methods based on the assumption of arbitrary missing patterns and thus used Markov chain Monte Carlo methods. This assumes multivariate normality to impute missing values (11.7%, 27/230 of the Web group and 12.5%, 18/144 of the print group were missing at follow-up). All analyses followed procedures described in the SAS OnlineDoc Version 8 for multiple imputation [24]. Logistic regression models were created to assess the impact of a priori determined predictor variables on the primary outcome—smoking status at follow-up. Variables that were significant in these models were considered potential variables to be entered into a multivariable logistic model predicting smoking status at follow-up. Any a priori determined variable (such as study site and depression level) were included in the final model selection regardless of bivariate significance. A parsimonious model that made clinical sense was developed by a process of forward and backward stepwise regression based on the individual variable significance in the model and the effect its removal had on other variable coefficients in the model. This was followed by assessing potential interaction effects, effect modifiers, and confounders. For the secondary outcome variables—quit attempts and readiness to quit smoking—similar model development occurred using polytomous logistic regression models with categorical outcome. All analyses were conducted in SAS Version 9.3.

## Results

### Participant Characteristics

**Figure 2:**
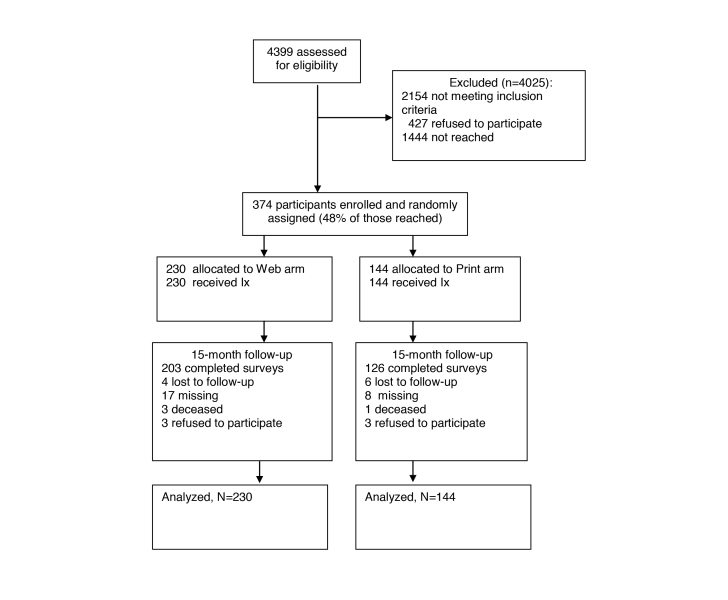
Recruitment and retention rates (Consolidated Standard of Reporting Trials Statement - CONSORT).

We assessed eligibility among 4399 survivors; 4025 people were excluded (35.88%, 1444/4025 were not reached; the majority of the remainder were ineligible due to smoking status); 46.7% (374/801) of eligible survivors were enrolled in the study. In total, 88.0% (329/374) of enrollees completed the 15-month follow-up (see Figure 2). In Figure 2, “lost to follow-up” were subjects for which no contact information was available at the 15-month time period whereas “missing” were those subjects who did not respond to the survey after multiple contact attempts.

Mean age at enrollment was 32 years (SD 7.94) and at cancer diagnosis was 12 years (SD 8.06). The sample was evenly split by gender (51.3%, 192/374 male) and was predominantly white (86.4%, 323/374). In total, 36.1% (135/374) had a high school education or less and 29.7% (111/374) had at least a college degree. About half (176/374) were married or living with a partner and the majority (79.4%, 297/374) were employed in the last year. There were no differences between the two arms at baseline on any demographic or cancer-related variables (see Table 1).

A total of 59.9% (224/374) of the sample smoked less than one pack of cigarettes per day (range <1 to 60; mean 10/day). About half of the sample was nicotine dependent. The majority (63.1%, 236/374) were in preparation to quit smoking and 55.9% (209/374) screened positive for possible depression.

There were no differences in any baseline demographic variables among completers and drop-outs except employment status; drop-outs were more likely to be employed.

### Primary Outcomes—Smoking Cessation

There were no significant differences between the two interventions arms in terms of smoking status at follow-up. At the final assessment, 16.5% of Web participants (22/132) and 15.5% of print participants (20/127) reported being abstinent for the previous 30 days (see Table 2).

Several factors were associated with smoking abstinence in multivariate analyses, including gender (*P*≤.04, males less likely to have quit at follow-up) and cancer treatment factors (higher cessation rates were associated with a cancer diagnosis of leukemia [*P*=.03] or non-Hodgkin’s lymphoma [*P*=.01], not having received radiation [*P*=.01], and having received surgery [*P*=.01]). Higher levels of perceived vulnerability for serious illness in the future was marginally associated with lower rates of cessation (*P*=.08 for more likely vulnerable) and less restrictive rules about smoking in the home were associated with significantly lower rates of cessation (*P*=.05).

An exploratory comparison of quit rates in the original PFH peer-delivered telephone intervention with the current Web and print condition suggests that the PFH-2 interventions attained equivalent levels of cessation. The original telephone-delivered intervention resulted in quit rates of 15% at 12-month follow-up. Although this was not a randomized comparison, these results do suggest that both Web- and print-based intervention methods developed specifically for cancer survivors do have a similar level of intervention impact to that found with a more intensive peer-counseling telephone intervention.

### Secondary Outcomes—Quit Attempts and Readiness to Change

There were no significant differences between the two arms in terms of quit attempts (see Table 3). On average, 35.1% (91/259) of participants made no quit attempts, 37.8% (98/259) made limited (1-3) attempts, and 27.0% (70/259) made extensive (4+) quit attempts over the 15-month follow-up period.

Demographic factors associated with quit attempts in multivariable analyses included male gender (*P*.001 and *P*=.06 for limited and extensive quit attempts, respectively), having less education (*P*=.01 for limited quit attempts), using pharmacotherapy (*P*<.001), being a more frequent computer user (*P*=.03-.06), perceiving a moderate (*P*=.03) or high level of vulnerability (*P*=.07) to serious future illness (for limited quit attempts), and not being nicotine dependent (*P*=.002 for extensive attempts).

There were also no significant differences between the two arms in terms of impact on readiness to quit smoking. Only two variables, using pharmacotherapy and nicotine dependence, were associated with readiness to quit in multivariable analyses: using pharmacotherapy was associated with being in the action (OR 5.33, 95% CI 1.20-23.64) (*P*=.03) and preparation stage (OR 6.05, 95% CI 1.81-20.17) (*P*=.01) (vs precontemplator). Those who were not nicotine dependent were more likely to be in later stages at the follow-up (OR 4.27, 95% CI 1.21-12.95) (*P*≤.01) and preparation versus precontemplation (OR 2.03, 95% CI 1.02-4.02) (*P*=.006).

**Table 1:** Baseline variables by treatment condition (n=329).

			Treatment condition				*P* value^a^
			Print (n=128)		Web (n=201)		
			n	%	n	%	
**Demographics**							
	Gender (Female)		70	55.6	93	45.8	.19
	**Education**						.85
		≤ high school or GED	43	34.1	75	36.9	
		Some college or training after college	50	39.7	63	31.0	
		College graduation	35	27.8	63	31.0	
	Age (LS means)		33.59		32.50		.23
	Employed, past year		97	77.0	159	78.3	.46
	**Race**						.47
		White	111	88.1	173	85.2	
		Non-white	17	13.5	28	13.8	
	**Marital status**						.71
		Married/living with partner	61	48.4	100	49.3	
		Never married and not living with partner	55	43.7	83	40.9	
		Divorced/no longer living with partner	12	9.5	18	8.9	
	**Cancer diagnosis**						.79
		Leukemia	34	27.0	45	22.2	
		Hodgkins disease	21	16.7	40	19.7	
		CNS malignancy	15	11.9	17	8.4	
		Non-Hodgkins Lymphoma	6	4.8	14	6.9	
		Bone cancer	10	7.9	15	7.4	
		Other	42	33.3	70	34.5	
		Received radiation	81	64.3	122	60.1	.71
		Received chemotherapy	96	76.2	153	75.4	.91
		Received surgery	93	73.8	141	69.5	.53
**Psychological variables**							
	**Spent at least part of the day worried about getting cancer again in past week**						.13
		Yes	22	17.5	24	11.8	
	**Impact of Events scale, Intrusive Thoughts subscale - categorized**						.22
		None	47	37.3	78	38.4	
		Little	22	17.5	47	23.2	
		Some	29	23.0	35	17.2	
		More	30	23.8	41	20.2	
	Screened positive for possible depression		85	67.5	100	49.3	.003
	Perceived personal control (LS means)		10.88		11.76		.07
	**Perceived risk for serious health problems in the future**						.61
		No chance/very unlikely/unlikely	29	23.0	41	20.2	
		Moderate chance	37	29.4	60	29.6	
		Likely	36	28.6	53	26.1	
		Very likely/certain to happen	25	19.8	44	21.7	
**Health**							
	**General health**						.67
		Excellent/very good	43	34.1	66	32.5	
		Good	53	42.1	77	37.9	
		Fair/poor	32	25.4	58	28.6	
	**Stage of change**						.86
		Precontemplation	15	11.9	33	16.3	
		Contemplation	34	27.0	39	19.2	
		Preparation	77	61.1	127	62.6	
**Computer Use**							
	**Computer use at baseline**						.79
		Daily	81	64.3	124	61.1	
		Less than daily, but monthly or more	20	15.9	43	21.2	
		Less than monthly or never	27	21.4	34	16.7	

^a^Controlling for site.

**Table 2:** Multivariable models predicting smoking at 15-month follow-up (n=374).

		Abstainer vs smoker at follow-up (adjusted for site)	
		*P* value	Adjusted OR (95% CI)
**Treatment condition**			
	Print	REF^a^	1.00
	Web	.87	1.07 (0.50-2.26)
**Gender**			
	Male	.04	0.49 (0.25-0.97)
	Female	REF	1.00
**Race**			
	White	.57	1.37 (0.46-4.06)
	Non-white	REF	1.00
**Screened positive for possible depression**			
	Yes	.22	0.62 (0.29-1.34)
	No	REF	1.00
**Diagnosis**			
	Leukemia	.03	2.64 (1.07-6.52)
	Hodgkins Disease	1.00	1.00 (0.31-3.20)
	CNS malignancy	.63	0.65 (0.12-3.68)
	Non-Hodgkins Lymphoma	.01	6.35 (1.48-27.17)
	Bone cancer	.86	1.13 (0.27-4.68)
	Other	REF	1.00
**Received radiation for CA**			
	Yes	.01	0.39 (0.19-0.81)
	No	REF	1.00
**Received surgery for CA**			
	Yes	.01	3.39 (1.29-8.88)
	No	REF	1.00
**Perceived vulnerability for serious illness in future**			
	No chance/very unlikely/unlikely	REF	1.00
	Moderate chance	.77	0.88 (0.37-2.11)
	Likely	.08	0.40 (0.14-1.12)
	Very likely/certain to happen	.12	0.45 (0.16-1.23)
**Rules about smoking in home at follow-up**			
	There are no rules	.05	0.38 (0.14-0.98)
	People can only smoke in certain rooms	.16	0.35 (0.08-1.53)
	People cannot smoke inside	REF	1.00

^a^REF: reference point.

**Table 3:** Multivariable models predicting quit attempts among smokers at 15-month follow-up (n=374).

			4 or more times vs none (adjusted for site)				1-3 times vs none (adjusted for site)			
			*P* value			Adjusted OR (95% CI)	*P* value			Adjusted OR (95% CI)
**Treatment condition**										
	Web		.90			1.05 (0.51-2.18)	.96			1.02 (0.54-1.90)
	Print		REF^a^			1.00	REF			1.00
**Gender**										
	Male		.06			2.06 (0.98-4.33)	.01			2.27 (1.18-4.40)
	Female		REF			1.00	REF			1.00
**Race**										
	White		.09			0.38 (0.12-1.18)	.01			0.28 (0.10-0.74)
	Non-white		REF			1.00	REF			1.00
**Screened positive for possible depression**										
	Yes		.30			0.67 (0.32-1.43)	.60			0.83 (0.43-1.63)
	No		REF			1.00	REF			1.00
**Education**										
		Did not complete HS / GED		.78	1.34 (0.16-11.36)			.01	5.24 (1.50-18.33)	
		Completed HS / GGED		.23	0.53 (0.19-1.49)			.58	0.77 (0.31-1.93)	
		Some college or training after HS		.79	1.13 (0.45-2.85)			.64	1.23 (0.51-2.97)	
		College graduate		REF	1.00			REF	1.00	
**Use medication to help quit smoking at follow-up**										
	Yes		<.001			9.45 (2.90-30.74)	<.001			9.27 (3.04-28.28)
	No		REF			1.00	REF			1.00
**Computer use**										
	Daily		.03			3.33 (1.12-9.90)	.06			2.20 (0.97-4.96)
	< than daily, but monthly or more		.03			4.01 (1.12-14.40)	.04			2.89 (1.05-7.95)
	< than monthly/never		REF			1.00	REF			1.00
**Experience any serious illness in future**										
	No chance/very unlikely/unlikely		REF			1.00	REF			1.00
	Moderate chance		.24			1.93 (0.64-5.80)	.03			3.33 (1.16-9.56)
	Likely		.36			1.67 (0.55-5.10)	.07			2.52 (0.91-6.97)
	Very likely/certain to happen		.52			0.69 (0.22-2.17)	.55			1.39 (0.47-4.09)
**Smoke within 30 minutes of waking**										
	Yes		REF			1.00	REF			1.00
	No		.002			3.70 (1.66-8.25)	.15			1.65 (0.83-3.26)

^a^REF: reference point.

### Process Outcomes

#### Intervention Use

Of the Web participants, 58.3% (77/132) logged on at least once (mean visits 3.25). Among visitors to the site, 13% (10/77) logged on once, 20% (15/77) twice, 13% (10/77) three times, and 54% (42/77) logged on four or more times (range 6-98 times). Those who reported using the website more frequently (3+ times) had higher quit attempts and lower smoking rates at follow-up, compared with less frequent users (see Table 4). We explored abstinence as an outcome related to different levels of use between groups; however, due to the distribution of quitters across intervention groups and use patterns, attempts to model the relationships were unstable.

Among the print condition participants, 58.3% (74/127) reported reading most or all of the print materials. About half reported using the materials on multiple occasions. Those who used more of the print materials (most/all) had higher quit attempts and lower smoking rates at follow-up, compared with those who used the materials less.

In total, 13.9% (36/259) of participants requested pharmacotherapy, with no differences between arms. Among those who requested this assistance, it was provided in 87% (31/36) of the cases; the primary reason for not providing it was that the participant did not have a regular physician who could confirm that there were no contraindications.

#### Satisfaction With Intervention

Of the Web participants who logged in, 87.9% (116/132) reported being satisfied or very satisfied with the site. Further, 75.8% (100/132) reported that the site provided new information about smoking, 83.3% (110/132) felt it provided new information about survivorship, and the majority felt that it was updated often enough; 81.1% (107/132) would recommend the site to other survivors.

In the print condition, 92.1% (117/127) reported being satisfied or very satisfied with the materials. Further, 93.7% (119/127) reported that the materials provided new information about smoking and 89.0% (113/127) felt they provided new information about survivorship; 88.2% (112/127) would recommend the materials to other survivors.

#### Access to the Web Intervention

In total, 79.9% (299/374) of the sample owned a computer and had access to the Internet at home and/or work; the majority (77.0%, 288/374) used the Internet at least once per week. Those who did not have regular Internet access had less education (*P*=.001), were more likely to be unemployed (*P*=.001), divorced (*P*=.001), and to be heavier smokers (*P*=.003). We offered MSN TV to those who did not have regular Internet; roughly two-thirds declined it.

**Table 4:** Quit attempts and smoking rate at follow-up among high/low intervention users.

	Quit Attempts		Smoking Rate (cigs/day)	
	Print	Web	Print	Web
Low Use	2.0	3.42	13.7	9.8
High Use	4.45	6.47	9.84	3.42

## Discussion

### Principal Findings

This paper assesses the efficacy of a print versus Web version of the Partnership for Health intervention. Both interventions were developed to address scalability of smoking cessation interventions among childhood and young adult cancer survivors. PFH-2 was designed to translate core components of PFH [9] to more scalable formats, and to determine whether equivalent levels of cessation could be achieved via a website tailored to cancer survivorship. Although we did indeed find that the PFH-2 intervention achieved similar levels of cessation to the peer counseling intervention, contrary to our hypothesis, there were no differences in cessation rates in the print versus Web arms and no differences in quit rates or changes in readiness to quit. Both interventions were viewed as substantive and appealing and were relatively comparable in terms of intervention “dose” based on participant report of use. These findings suggest that either the print or Web-format intervention could be recommended for survivors who smoke, as these cessation interventions yield equivalent levels of success to those found in our previous telephone-delivered intervention. The outcomes are also comparable to other Internet treatment studies [25] and PHS clinical guidelines for cost-effective interventions.

A Web-based approach was selected because of the presumed computer affinity of this younger population, as well as the potential for dissemination. We did offer access to the Internet via MSN TV to the 20% that did not have Internet access. Surprisingly, the majority of these participants declined the offer. This may reflect an active choice among these individuals not to engage with this technology and suggests that, at least in the population of childhood and young adult cancer survivors, efforts to increase access may not be helpful. Among those who received the print materials, engagement was good, which may be preferable in some settings and to some participants.

A key issue facing any disseminated, patient-guided intervention is level of patient engagement. Low levels of intervention use is a common problem in both Web and print interventions [26-30] and rapid and consistent declines in intensity of use occur over time [31,32]. A dose-response between website use and abstinence outcomes has been documented [33-35]. Consistent with this literature, in PFH-2 use of the website was associated with cessation-related efforts. Chiu and Eysenbach [36] call for development of a research agenda targeting how to improve use of Web-based interventions, which is critical if they are to be helpful in taking more labor-intensive interventions to scale. The increasing penetration of social media and technology may present opportunities for increasing engagement, as noted by Graham et al [33,37]. Active participation in online communities is associated with higher rates of cessation, and integrating smokers into an online social network can increase support and may also increase utilization of cessation tools and nicotine replacement therapy [38]. Text messages and Twitter could be utilized as efficient ways for peer counselors to engage survivors. The increased access to video cameras on laptops and smartphones also provides opportunities to evaluate Skype and videoconferencing as engagement strategies, although these strategies may also present issues when taken to scale. In the present study, we targeted all smokers—not just those who were interested in quitting smoking. The intervention was designed and presented with a focus on general survivorship issues, in addition to smoking, and was organized by stage of readiness to change. That said, those with less interest in cessation would be expected to have lower levels of engagement with cessation content, although integrating smoking-related information into other forms of interventions or materials for survivors may be a way to increase their reconsideration of smoking.

Use of pharmacotherapy was quite low, particularly given the high level of readiness to quit and the fact that it was available at no cost. This is somewhat puzzling, but may be a function of survivors’ heavy use of medications as part of cancer treatment and possibly survivorship, which may make them more reticent to use pharmacologic treatments for non-cancer-related issues. In 2014, CMS (Center for Medicare and Medicaid Services) programs will cover all FDA-approved medications and cessation counseling [39]. There are very tangible and important health benefits associated with population-level access to evidence-based cessation treatments in the general population [40-42], and the benefits for survivors may be even greater, given the synergistic effects between smoking and their increased risk of late effects. Our findings suggest that while access is important, alone it may not be sufficient in this high-risk population to achieve large-scale increases in pharmacotherapy use. This is an area that warrants further research attention.

This study highlights the need to develop an effective infrastructure for delivery of smoking cessation services to childhood and young adult cancer survivors. The infrastructure for identifying smokers within long-term survivorship care programs is largely missing [11] and a more systematic approach to patient tracking and follow-up is needed. Survivorship programs should be strongly encouraged to follow the PHS guidelines for delivery of smoking cessation services in clinical care settings [43,44]. Given the current movement toward electronic health records [45], it is likely that there will be greater opportunity for developing this infrastructure within survivorship programs. The American Society of Preventive Oncology recently called for a paradigm change related to cancer prevention after cancer, highlighting a critical need to reduce preventable risk factors [46]. Tobacco use is not systematically assessed in US cancer programs—less than half of comprehensive cancer centers have a strategy in place for effective identification of tobacco use [47] and only 28% use any tobacco-related quality improvement measures. As a result, a large proportion of smokers with cancer do not receive formal assistance with quit attempts. An important question would be whether survivorship-focused interventions are needed or if survivors could be integrated into widely available Internet-based cessation programs, such as QuitNet or BecomeAnEx*.* Our previous qualitative work suggests that the survivorship identity may be quite important in terms of being willing to engage in smoking cessation [48]. However, additional experimental work to determine the added value of tailoring to survivorship would be extremely valuable.

Cancer survivors are a unique population that should be uniquely aware of risks for cancer. However, not all childhood and young adult survivors are well informed about their cancer, its treatment, and the associated late effects, in large part because of the young age at diagnosis, disease, and treatment complexity, and the fact the knowledge about late effects has been emerging over time [49,50]. The Childhood Cancer Survivor Study has demonstrated that emotional distress is related to health behaviors, including smoking in long-term survivors, and subgroups that are at risk for becoming smokers (eg, lower income survivors, those with multiple medical morbidities) have higher distress levels [51,52]. A study of adolescent and young adult survivors did not find statistically significant differences between survivors and controls in terms of psychological distress or health-related quality of life, but survivors had less positive health beliefs [53]. Of note, these survivors’ beliefs reflected their perceived bad luck and uncertainty about their health, including concerns about future medical problems (ie, health perceptions) and beliefs (ie, cognitive competence) that they have cognitive challenges that could impact their function (eg, memory, attention, intellect). These types of beliefs could impact on positive coping, such as smoking cessation.

The lack of an effective implementation infrastructure posed a challenge in the design of PFH-2, as it does in many implementation research studies. We considered comparing the PFH-1 peer counseling condition with the website. However, if we had concluded that the peer counseling was superior, we would not have moved the field forward in terms of having an evidence-based intervention that could be sustained and scaled in real-world conditions. Given that we did not have a mechanism to deploy peer counselors that would be sustainable following completion of the study, we felt it was best to learn from the peer counselor model and adapt to a sustainable format. Thus, PFH-2 was designed with the real-world constraints of survivorship care in mind and compared best survivorship-focused print materials with the website, which provided some interactivity but was more sustainable and scalable than peer counseling. This approach is consistent with several of the broad principles outlined by Kottke et al [54], which are necessary in order for health research to have a greater impact on patient and population health outcomes, including: (1) the needs of patients and populations determine the research agenda, (2) the research agenda addresses contextual and implementation issues, including the development of delivery and accountability systems, and (3) the research agenda determines the research methods rather than the methods determine the research agenda. Kottke and colleagues also note that the goal should be to optimize practice through research, which was the approach we took in designing PFH-2. This approach also addresses significant concerns raised within implementation science about having an increased emphasis on external validity and moving away from artificial comparisons that have little bearing on real-world care delivery [55-58]. In the interests of maximizing fidelity and ensuring a minimum intervention dose delivered, we did provide print materials to participants in the Web condition who did not access the website after 3 months. Although this means that the Web condition was not “pure”, as some participants had access to both the Web and print interventions, we felt that ethically it was important to ensure that patients received the intervention content in some form, when we knew that participants had not received it via the Web.

### Limitations

Study limitations should be noted. The response rate was impacted by stringent IRB requirements regarding patient contact and release of contact information to the coordinating center, which may impact on generalizability of findings. Cessation outcomes were self-reported, which is typical in population-level and Web-based studies such as this, but still a limitation [59]; the bogus pipeline procedure, a well-accepted strategy for increasing the accuracy of self-report, was used [17,18]. It is possible that self-report at the point of evaluation of study eligibility introduced a sampling bias. However, participants were not aware of the eligibility requirements at the time of recruitment, which minimized the likelihood of bias. Further, smokers who did not accurately report their smoking status would likely report this same inaccuracy in the context of their health care, and thus would avoid exposure to this type of intervention. Therefore, any reporting bias would not likely effect the outcome evaluation.

There are several important strengths to note. A population-based approach was used in conducting this study, identifying all potential smokers within several different survivorship programs in the United States and Canada, which contributes to the external validity of the findings. Data were conservatively analyzed using multiple imputation methods for missing data. This study builds on the previous effective PFH intervention and was designed to determine how best to deliver that intervention in a more scalable format. The study design emphasized external validity and maximizing generalizability of study findings.

### Conclusions

Smoking cessation among childhood and young adult cancer survivors is critical. Effective evidence-based programs should be integrated into primary and survivorship care delivery on an on-going and routine basis. This study demonstrated that it was possible to achieve equivalent cessation rates with tailored and targeted Web- and print-based materials designed specifically for survivors and that these cessation rates were equivalent to those found with a more labor-intensive telephone-based intervention. These findings suggest that survivorship programs have flexibility in the format in which cessation services are delivered without sacrificing effectiveness. Patients who reported more frequent use of the materials had better cessation rates, suggesting that developing methods for increasing patient engagement in print and Web-based interventions might optimize outcomes. Future research should examine such approaches to increase engagement and should also evaluate whether survivorship-focused interventions are critical or if survivors experience equal benefits from robust Web-based interventions available to the general public.

## Multimedia Appendix 1

CONSORT-EHEALTH checklist V1.6.2 [60].

## Abbreviations

BFR: baseline feedback report
IES: Impact of Events Scale
IRB: Institutional Review Board
PFH: Partnership for Health
PFH-2: Partnership for Health-2
PHS: Public Health Service
