# Supplementary material for: Partnership for Health-2, A Web-Based Versus Print Smoking Cessation Intervention for Childhood and Young Adult Cancer Survivors: Randomized Comparative Effectiveness Study
Source: J Med Internet Res. 2013 Nov 5;15(11):e218. doi: 10.2196/jmir.2533 (PMC3841363; doi:10.2196/jmir.2533)
Supplement: Supplementary file 1 [file jmir_v15i11e218_app1.pdf]

CONSORT-EHEALTH Checklist V1.6.2 Report

(based on CONSORT-EHEALTH V1.6), available at [http://tinyurl.com/consort-ehealth-v1-6].

2533

Date completed

1/16/2013 16:44:05

by

karen emmons

A Randomized Comparative Effectiveness Study of A Web-Based vs Print
Smoking Cessation Intervention for Childhood and Young Adult Cancer Survivors: The Partnership for Health-2 Study

TITLE

1a-i) Identify the mode of delivery in the title

"A Randomized Comparative Effectiveness Study of A Web-Based vs Print
Smoking Cessation Intervention for Childhood and Young Adult Cancer Survivors: The Partnership for Health-2 Study"

1a-ii) Non-web-based components or important co-interventions in title

"A Randomized Comparative Effectiveness Study of A Web-Based vs Print
Smoking Cessation Intervention for Childhood and Young Adult Cancer Survivors: The Partnership for Health-2 Study"

1a-iii) Primary condition or target group in the title

"A Randomized Comparative Effectiveness Study of A Web-Based vs Print
Smoking Cessation Intervention for Childhood and Young Adult Cancer Survivors: The Partnership for Health-2 Study"

ABSTRACT

1b-i) Key features/functionalities/components of the intervention and comparator in the METHODS section of the ABSTRACT

"This article reports on Partnership for Health-2 (PFH-2), an effort to develop an effective and disseminable version of Partnership for Health (PFH), a previously tested peer-delivered telephone counseling program that doubled smoking cessation rates among childhood cancer survivors who smoke. "

"Participants were randomly assigned to a web-based or print format of the PFH intervention; all had access to free pharmacotherapy. The website was designed to provide new content at each log-on, and a peer counselor moderator a forum/chat feature."

1b-ii) Level of human involvement in the METHODS section of the ABSTRACT

"The website was designed to provide new content at each log-on, and a peer counselor moderator a forum/chat feature. "

1b-iii) Open vs. closed, web-based (self-assessment) vs. face-to-face assessments in the METHODS section of the ABSTRACT

"The primary outcome was smoking status at 15 months post-randomization, assessed via telephone survey (using bogus pipeline procedure to improve self-report)."

1b-iv) RESULTS section in abstract must contain use data

"This study was a randomized controlled trial with a 15-month follow-up that included 374 smokers who were survivors of childhood or young adult cancers, recruited from five survivorship clinics."

"Fifty-eight percent of web participants logged on at least once (M visits = 3.25)."

1b-v) CONCLUSIONS/DISCUSSION in abstract for negative trials

The print and web-formats yielded equivalent levels of success to those found with a telephone-delivered intervention. This study provides important options for survivorship programs that may not have resources for interpersonal forms of cessation counseling. Efforts to increase patient use of the interventions may result in higher cessation rates."

INTRODUCTION

2a-i) Problem and the type of system/solution

"This team's previous work has demonstrated the efficacy of Partnership for Health (PFH), a survivor-focused peer-delivered telephone counseling intervention for smoking cessation. PFH led to a doubling in quit rates compared with usual care, and the intervention effect was sustained over two to five years of follow-up (Emmons et al. 2005 ; Emmons et al. 2009). The connection that the peer-delivered intervention provided between survivors was an important way to engage participants in the intervention. However, it is challenging to scale interventions that include on-going counseling. "

"This paper presents results from a randomized control trial evaluating the effectiveness of Partnership for Health-2 in targeted and tailored web-based versus print formats. The overall goal is to determine if the intervention outcomes in these self-guided, scalable formats approximate what was found in a more intensive, telephone counseling program."

2a-ii) Scientific background, rationale: What is known about the (type of) system

"Remarkable improvements in the treatment and long-term survival of childhood and young adult (c/ya) cancer survivors have resulted in prevention of adverse late effects and second primary cancers being a key part of survivorship care (Friedman et al. 2010; Hewitt et al. 2003; Meacham et al. 2009; Mulrooney et al. 2009; Ries et al. 2007).   Smoking rates among this population are substantial (Emmons et al. 2002; Emmons et al. 2003; Frobisher et al. 2008).

This team's previous work has demonstrated the efficacy of Partnership for Health (PFH), a survivor-focused peer-delivered telephone counseling intervention for smoking cessation. PFH led to a doubling in quit rates compared with usual care, and the intervention effect was sustained over two to five years of follow-up (Emmons et al. 2005 ; Emmons et al. 2009). The connection that the peer-delivered intervention provided between survivors was an important way to engage participants in the intervention. However, it is challenging to scale interventions that include on-going counseling.

A recent evaluation of existing infrastructure for delivering smoking cessation services in the context of survivorship programs revealed relatively few resources; only 3% of programs assessed smoking status at every visit, as recommended by Public Health Service guidelines, and only 25% offered cessation services (de Moor et al. 2007). Further, cancer survivors are quite geographically dispersed, and thus effective interventions are needed that can be easily scaled and delivered remotely regardless of survivors' location. The present study focused on the adaptation of the PFH peer-delivered intervention for a web- and print-based format as a way to increase the intervention's dissemination potential and sustainability. We selected web- and print-based interventions because of the relatively high penetration of internet access in the target age group, and because these formats could be easily integrated into standard practice at survivorship programs across the country, compared with telephone-based interventions."

METHODS

3a) CONSORT: Description of trial design (such as parallel, factorial) including allocation ratio

"The overall goal is to determine if the intervention outcomes in these self-guided, scalable formats approximate what was found in a more intensive, telephone counseling program. Our hypothesis was that the outcomes of the web and print formats would be equivalent, and would be similar to that found in the original PFH counseling intervention."

3b) CONSORT: Important changes to methods after trial commencement (such as eligibility criteria), with reasons

"Original sample size calculations were adjusted due to the discovery during implementation that the participating survivorship programs' estimates of smokers were outdated."

3b-i) Bug fixes, Downtimes, Content Changes

N/A

4a) CONSORT: Eligibility criteria for participants

"Eligibility included: diagnosed with cancer before age 35, currently between ages 18-55, completed cancer treatment for ≥ 2 years, mentally able to provide informed consent, reachable by telephone, able to speak English, and are a current smoker (defined as smoking within the previous 30 days). Participants were not required to be interested in smoking cessation in order to participate."

4a-i) Computer / Internet literacy

N/A.

4a-ii) Open vs. closed, web-based vs. face-to-face assessments:

"PFH-2 was conducted in collaboration with five cancer centers in the United States and Canada, with Institutional Review Board (IRB) approval at all sites. The study was also advertised on childhood and young adult survivorship Web sites. Eligibility included: diagnosed with cancer before age 35, currently between ages 18-55, completed cancer treatment for ≥ 2 years, mentally able to provide informed consent, reachable by telephone, able to speak English, and are a current smoker (defined as smoking within the previous 30 days). Participants were not required to be interested in smoking cessation in order to participate. Baseline data collection began on December 2005, and follow-up data collection ended in October 2009.

A preliminary screen for eligiblity was performed at each site via medical record review or brief telephone screening. After consent for sharing contact information was obtained, contact information was forwarded to the survey team to verify eligibility, obtain informed consent for study participation, and administer the baseline telephone survey."

4a-iii) Information giving during recruitment

"Participants were informed that the study was examining different ways to deliver health information, including information about tobacco use, to survivors. They were not required to be interested in smoking cessation in order to participate."

4b) CONSORT: Settings and locations where the data were collected

"A preliminary screen for eligiblity was performed at each site via medical record review or brief telephone screening. After consent for sharing contact information was obtained, contact information was forwarded to the survey team to verify eligibility, obtain informed consent for study participation, and administer the baseline telephone survey."

4b-i) Report if outcomes were (self-)assessed through online questionnaires

"After consent for sharing contact information was obtained, contact information was forwarded to the survey team to verify eligibility, obtain informed consent for study participation, and administer the baseline telephone survey."

"The intervention period was 6 months, and a follow-up survey was completed by telephone at 15 months after randomization. The bogus pipeline procedure was used to increase accuracy of self-reported smoking status (Murray et al. 1987; Murray and Perry 1987). The survey team that collected the outcome data was blind to condition assignment."

4b-ii) Report how institutional affiliations are displayed

Participants were recruited through their treating cancer center. From there forward, the study was branded as "Partnership for Health".

5) CONSORT: Describe the interventions for each group with sufficient details to allow replication, including how and when they were actually administered

5-i) Mention names, credential, affiliations of the developers, sponsors, and owners

We developed the intervention internally. There are not conflicts. The original intervention received a SAMSHA award, and is registered on the HHS' Research Tested Programs (RTPs) website.

5-ii) Describe the history/development process

The website was developed from the original PFH intervention. We did extensive usability testing and pilot testing prior to launch.

5-iii) Revisions and updating

"Following completion of the baseline survey, those assigned to the Web intervention were sent log-on information. The web intervention consisted of seven discrete tailored sessions designed to parallel the counseling sessions of the original PFH study and mirror the basic content the PFH2 print materials. The content was dynamically tailored, matching the participants' stage of readiness. Upon first log on, participants saw their BFR, described above, on their home page. The home page also highlighted active components of the intervention that participants had not yet navigated. Patients saw refreshed content on the home page as they progressed from session to session. A peer counselor moderated the web site's discussion forum, and served as a resource for questions."

5-iv) Quality assurance methods

We maintained several test accounts for quality assurance purposes.

5-v) Ensure replicability by publishing the source code, and/or providing screenshots/screen-capture video, and/or providing flowcharts of the algorithms used

Screenshots are included in the manuscript. We will provide source code as requested.

5-vi) Digital preservation

Although the website is archived internally, the website, if used in the future, would need to be updated.

5-vii) Access

"Following completion of the baseline survey, those assigned to the Web intervention were sent log-on information. "

"We offered MSNTV to those who did not have regular internet access."

5-viii) Mode of delivery, features/functionalities/components of the intervention and comparator, and the theoretical framework

"Both conditions included key features of the original PFH peer-delivered intervention, including a letter encouraging smoking cessation from the site oncologist and free pharmacotherapy (nicotine patch or Zyban) for themselves and any smoking partner/spouse that wished to quit. Those who were interested in pharmacotherapy contacted study staff and provided permission to contact their health care provider for approval using a fax back for. We also the USDHHS/AHCPR pamphlet “Helping Smokers Quit: A guide for primary care clinicians” and a basic fact sheet about adult survivors of c/ya cancer. Pharmacotherapy was sent to the doctor’s office for distribution to the patient.

The Print Materials arm received tailored and targeted self help materials. A baseline feedback report (BFR) was generated for each individual, reflecting information patients gave on the baseline about their readiness to quit, perceived risk, nicotine dependence, and based on these factors, which intervention manual to start with. The BFR also gave basic facts about cancer treatment, and illustrated how cancer, cancer treatment, and smoking affects many of the same organs (see Figure 2). Each manual addressed participant-specific barriers to change, organized by level of readiness to change, and other survivor-related topics of interest. Testimonials and stories of other survivors’ experiences were used to provide the survivor-to-survivor connection. The Materials condition was designed to reflect as many of the realities as possible of how the intervention might be utilized when scaled to existing cancer survivorship clinics.

PFH-2 Web Intervention:
Following completion of the baseline survey, those assigned to the Web intervention were sent log-on information. The web intervention consisted of seven discrete tailored sessions designed to parallel the counseling sessions of the original PFH study and mirror the basic content the PFH2 print materials. The content was dynamically tailored, matching the participants’ stage of readiness. Upon first log on, participants saw their BFR, described above, on their home page. The home page also highlighted active components of the intervention that participants had not yet navigated. Patients saw refreshed content on the home page as they progressed from session to session. A peer counselor moderated the web site’s discussion forum, and served as a resource for questions.

Participants who never logged in or stopped using the site received additional email prompts that highlighted content that survivors might find particularly important or engaging, along with bi-weekly emailed newsletters. Those who had not accessed the website within 11 weeks were sent a final letter along with the print materials to increase the likelihood of some exposure to the intervention content, and to approximate likely approaches in a clinic setting. Participants had access to the website for six months regardless of their log-in status. For quality assurance purposes, several “test participants” were created and followed throughout the implementation period, to identify glitches or issues with the web system."

5-ix) Describe use parameters

"The web intervention consisted of seven discrete tailored sessions designed to parallel the counseling sessions of the original PFH study and mirror the basic content the PFH2 print materials. The content was dynamically tailored, matching the participants’ stage of readiness. Upon first log on, participants saw their BFR, described above, on their home page. The home page also highlighted active components of the intervention that participants had not yet navigated. Patients saw refreshed content on the home page as they progressed from session to session. A peer counselor moderated the web site’s discussion forum, and served as a resource for questions.

Participants who never logged in or stopped using the site received additional email prompts that highlighted content that survivors might find particularly important or engaging, along with bi-weekly emailed newsletters. Those who had not accessed the website within 11 weeks were sent a final letter along with the print materials to increase the likelihood of some exposure to the intervention content, and to approximate likely approaches in a clinic setting. Participants had access to the website for six months regardless of their log-in status. For quality assurance purposes, several “test participants” were created and followed throughout the implementation period, to identify glitches or issues with the web system."

5-x) Clarify the level of human involvement

"The web intervention consisted of seven discrete tailored sessions designed to parallel the counseling sessions of the original PFH study and mirror the basic content the PFH2 print materials. The content was dynamically tailored, matching the participants’ stage of readiness. Upon first log on, participants saw their BFR, described above, on their home page. The home page also highlighted active components of the intervention that participants had not yet navigated. Patients saw refreshed content on the home page as they progressed from session to session. A peer counselor moderated the web site’s discussion forum, and served as a resource for questions.

Participants who never logged in or stopped using the site received additional email prompts that highlighted content that survivors might find particularly important or engaging, along with bi-weekly emailed newsletters. Those who had not accessed the website within 11 weeks were sent a final letter along with the print materials to increase the likelihood of some exposure to the intervention content, and to approximate likely approaches in a clinic setting. Participants had access to the website for six months regardless of their log-in status. For quality assurance purposes, several “test participants” were created and followed throughout the implementation period, to identify glitches or issues with the web system."

5-xi) Report any prompts/reminders used

"Participants who never logged in or stopped using the site received additional email prompts that highlighted content that survivors might find particularly important or engaging, along with bi-weekly emailed newsletters. Those who had not accessed the website within 11 weeks were sent a final letter along with the print materials to increase the likelihood of some exposure to the intervention content, and to approximate likely approaches in a clinic setting."

5-xii) Describe any co-interventions (incl. training/support)

"Both conditions included key features of the original PFH peer-delivered intervention, including a letter encouraging smoking cessation from the site oncologist and free pharmacotherapy (nicotine patch or Zyban) for themselves and any smoking partner/spouse that wished to quit. Those who were interested in pharmacotherapy contacted study staff and provided permission to contact their health care provider for approval using a fax back for. We also the USDHHS/AHCPR pamphlet “Helping Smokers Quit: A guide for primary care clinicians” and a basic fact sheet about adult survivors of c/ya cancer. Pharmacotherapy was sent to the doctor’s office for distribution to the patient. "

6a) CONSORT: Completely defined pre-specified primary and secondary outcome measures, including how and when they were assessed

" Logistic regression models were created to assess the impact of predictor variables on the primary outcome--smoking status at follow-up... For the secondary outcome variables -- quit attempts and readiness to quit smoking-- similar model development occurred..."

"Smoking behavior: Smoking status was assessed by self-reported assessment of smoking, even a puff, in the past 30 days. The bogus pipeline procedure was used to increase the accuracy of self-report."

"Quit attempts were assessed by the number of quits in the previous 12 months with at least 24 hours abstinence."

"Motivational Variables: The Stages of Change Scale assessed motivation to quit smoking (Prochaska and DiClemente 1993), according to four categories: (1) precontemplation: not seriously thinking about quitting in the next 6 months; (2) contemplation: seriously thinking about quitting in the next 6 months; (3) preparation: intending to quit in the next month and have tried to quit in the past year; and (4) action: not currently smoking and quit within the past six months."

6a-i) Online questionnaires: describe if they were validated for online use and apply CHERRIES items to describe how the questionnaires were designed/deployed
N/A
6a-ii) Describe whether and how "use" (including intensity of use/dosage) was defined/measured/monitored
"Intervention Use: Use of the web-based intervention was assessed using web analytics. Use of the print intervention was assessed on the follow-up survey, with questions about percentage of the materials read and frequency of use."
6a-iii) Describe whether, how, and when qualitative feedback from participants was obtained
Participants could provide qualitative feedback through the forum/chat room.
6b) CONSORT: Any changes to trial outcomes after the trial commenced, with reasons
N/A
7a) CONSORT: How sample size was determined
7a-i) Describe whether and how expected attrition was taken into account when calculating the sample size
"Original sample size calculations were adjusted due to the discovery during implementation that the participating survivorship programs' estimates of smokers were outdated. Thus, using a 5:3 allocation scheme, we had 71% power to detect a difference of 9% in quit rate between the web and print groups." Attrition was considered in the power calculations.
7b) CONSORT: When applicable, explanation of any interim analyses and stopping guidelines
N/A
8a) CONSORT: Method used to generate the random allocation sequence
"The random allocation sequence was generated by the study biostatistician. Randomization was done by the survey team, supervised by the biostatistician, following completion of the baseline survey."
8b) CONSORT: Type of randomisation; details of any restriction (such as blocking and block size)
"PFH-2 was a stratified randomized controlled trial with cancer center as strata."
9) CONSORT: Mechanism used to implement the random allocation sequence (such as sequentially numbered containers), describing any steps taken to conceal the sequence until interventions were assigned
"The random allocation sequence was generated by the study biostatistician. Randomization was done by the survey team, supervised by the biostatistician, following completion of the baseline survey."

"The survey team that collected the outcome data was blind to condition assignment."
10) CONSORT: Who generated the random allocation sequence, who enrolled participants, and who assigned participants to interventions
"The random allocation sequence was generated by the study biostatistician. Randomization was done by the survey team, supervised by the biostatistician, following completion of the baseline survey."
11a) CONSORT: Blinding - If done, who was blinded after assignment to interventions (for example, participants, care providers, those assessing outcomes) and how
11a-i) Specify who was blinded, and who wasn't
"The survey team that collected the outcome data was blind to condition assignment."
11a-ii) Discuss e.g., whether participants knew which intervention was the "intervention of interest" and which one was the "comparator"
They did not know which intervention was the comparator.
11b) CONSORT: If relevant, description of the similarity of interventions
"Both conditions included key features of the original PFH peer-delivered intervention, including a letter encouraging smoking cessation from the site oncologist and free pharmacotherapy (nicotine patch or Zyban) for themselves and any smoking partner/spouse that wished to quit. Those who were interested in pharmacotherapy contacted study staff and provided permission to contact their health care provider for approval using a fax back for. We also the USDHHS/AHCPR pamphlet "Helping Smokers Quit: A guide for primary care clinicians" and a basic fact sheet about adult survivors of c/ya cancer. Pharmacotherapy was sent to the doctor's office for distribution to the patient.

A baseline feedback report (BFR) was generated for each individual, reflecting information patients gave on the baseline about their readiness to quit, perceived risk, nicotine dependence, and based on these factors, which intervention manual to start with. The BFR also gave basic facts about cancer treatment, and illustrated how cancer, cancer treatment, and smoking affects many of the same organs (see Figure 2). Each manual addressed participant-specific barriers to change, organized by level of readiness to change, and other survivor-related topics of interest. Testimonials and stories of other survivors' experiences were used to provide the survivor-to-survivor connection. The Materials condition was designed to reflect as many of the realities as possible of how the intervention might be utilized when scaled to existing cancer survivorship clinics.

PFH-2 Web Intervention:
Following completion of the baseline survey, those assigned to the Web intervention were sent log-on information. The web intervention consisted of seven discrete tailored sessions designed to parallel the counseling sessions of the original PFH study and mirror the basic content the PFH2 print materials. The content was dynamically tailored, matching the participants' stage of readiness. Upon first log on, participants saw their BFR, described above, on their home page. The home page also highlighted active components of the intervention that participants had not yet navigated. Patients saw refreshed content on the home page as they progressed from session to session. A peer counselor moderated the web site's discussion forum, and served as a resource for questions.

Participants who never logged in or stopped using the site received additional email prompts that highlighted content that survivors might find particularly important or engaging, along with bi-weekly emailed newsletters. Those who had not accessed the website within 11 weeks were sent a final letter along with the print materials to increase the likelihood of some exposure to the intervention content, and to approximate likely approaches in a clinic setting."
12a) CONSORT: Statistical methods used to compare groups for primary and secondary outcomes
"Baseline comparisons of patient characteristics between intervention arms were assessed. Depression level was the only variable of significance in this comparison and was, in addition to site, controlled for in all future analyses. All outcome analyses were conducted using multiple imputation methods (in SAS MI) to account for missing data (Yuan Y 2011). Logistic regression models were created to assess the impact of predictor variables on the primary outcome--smoking status at follow-up. Variables that were significant in these models were entered into a multivariable logistic model predicting smoking status at follow-up. A parsimonious model was developed after assessing effect modifiers and confounders. For the secondary outcome variables -- quit attempts and readiness to quit smoking-- similar model development occurred using polytomous logistic regression models with categorical outcome. All analyses were conducted in SAS Version 9.3."
12a-i) Imputation techniques to deal with attrition / missing values
"All outcome analyses were conducted using multiple imputation methods (in SAS MI) to account for missing data (Yuan Y 2011)"
12b) CONSORT: Methods for additional analyses, such as subgroup analyses and adjusted analyses
N/A

RESULTS

13a) CONSORT: For each group, the numbers of participants who were randomly assigned, received intended treatment, and were analysed for the primary outcome

This is addressed in the Consort Figure that is included in the paper.

13b) CONSORT: For each group, losses and exclusions after randomisation, together with reasons

This is addressed in the Consort Figure that is included in the paper.

13b-i) Attrition diagram

We have provided data on use over the study period, but do not have the capability to compare attrition over time in use across the two study groups (we do not have over-time use data for print participants), and thus present the data over the study period only.

14a) CONSORT: Dates defining the periods of recruitment and follow-up

"Baseline data collection began on December 2005, and follow-up data collection ended in October 2009."

14a-i) Indicate if critical "secular events" fell into the study period

N/A

14b) CONSORT: Why the trial ended or was stopped (early)

N/A

15) CONSORT: A table showing baseline demographic and clinical characteristics for each group

There were no differences between the two arms at baseline on any demographic or cancer-related variables. Thus, we provide this data for the overall sample in the text.

15-i) Report demographics associated with digital divide issues

"Access to the Web Intervention: About 80% of the sample owned a computer and had access to the Internet at home and/or work; the majority (77%) used the Internet at least once per week. Those who did not have regular access had less education ($p<.001$), were more likely to be unemployed ($p<.001$), divorced ($p<.001$), and to be heavier smokers ($p<.003$). We offered MSNTV to those who did not have regular internet; about two-thirds declined it. "

"A Web-based approach was selected because of the presumed computer affinity of this younger population, as well as the potential for dissemination. We did offer access to the internet via MSN-TV to the 20% that did not have internet access. Surprisingly, the majority of these participants declined the offer. This may reflect an active choice among these individuals not to engage with this technology, and suggests that, at least in the population of c/ya cancer survivors, efforts to increase access may not be helpful. Among those who received the print materials, engagement was good, which may be preferable in some settings and to some participants."

16a) CONSORT: For each group, number of participants (denominator) included in each analysis and whether the analysis was by original assigned groups

16-i) Report multiple "denominators" and provide definitions

N's are provided in all tables.

16-ii) Primary analysis should be intent-to-treat

"Data were conservatively analyzed using intention-to-treat."

17a) CONSORT: For each primary and secondary outcome, results for each group, and the estimated effect size and its precision (such as 95% confidence interval)

This information is provided in the tables.

17a-i) Presentation of process outcomes such as metrics of use and intensity of use

"Process Outcomes
Intervention Use: Fifty-eight percent of Web participants logged on at least once (M visits = 3.25). Among visitors to the site, 13% logged on once, 20% twice, 13% three times, and 54% logged on four or more times (range: 6-98 times). Those who reported using the website more frequently (3+ times) had higher quit attempts and lower smoking rates at follow-up, compared with less frequent users (see Figure 3).

Among the print condition participants, 58% reported reading most or all of the print materials. About half reported using the materials on multiple occasions. Those who used more of the print materials (most/all) had higher quit attempts and lower smoking rates at follow-up, compared with those who used the materials less.

Fourteen percent of participants requested pharmacotherapy, with no differences between arms. Among those who requested this assistance, it was provided in 87% of the cases; the primary reason for not providing it was that the participant did not have a regular physician who could confirm that there were no contraindications.

Satisfaction with Intervention: Eighty-eight percent of web participants who logged in reported being satisfied or very satisfied with the site. Seventy-six percent reported that the site provided new information about smoking, 83% felt it provided new information about survivorship, and the majority felt that it was updated often enough; 81% would recommend the site to other survivors.
In the print condition, 92% reported being satisfied or very satisfied with the materials. Ninety-four percent reported that the materials provided new information about smoking, and 89% felt they provided new information about survivorship; 88% would recommend the materials to other survivors."

17b) CONSORT: For binary outcomes, presentation of both absolute and relative effect sizes is recommended

We have presented odds ratios. We did not calculate risk differences, but will add this if requested upon review.

18) CONSORT: Results of any other analyses performed, including subgroup analyses and adjusted analyses, distinguishing pre-specified from exploratory

N/A

18-i) Subgroup analysis of comparing only users

N/A

19) CONSORT: All important harms or unintended effects in each group

N/A

19-i) Include privacy breaches, technical problems

N/A.

19-ii) Include qualitative feedback from participants or observations from staff/researchers

N/A.

DISCUSSION

20) CONSORT: Trial limitations, addressing sources of potential bias, imprecision, multiplicity of analyses

20-i) Typical limitations in ehealth trials

"Study limitations should be noted. The response rate was impacted by stringent IRB requirements regarding patient contact and release of contact information to the coordinating center, which may impact on generalizability of findings. Cessation outcomes were self-reported, which is typical in population-level and web-based studies such as this, but still a limitation (Velicer et al. 1992); the bogus pipeline procedure, a well-accepted strategy for increasing the accuracy of self-report, was used (Murray et al. 1987; Murray and Perry 1987). It is possible that self-report at the point of evaluation of study eligibility introduced a sampling bias. However, participants were not aware of the eligibility requirements at the time of recruitment, which minimized the likelihood of bias. Further, smokers who did not accurately report their smoking status would likely report this same inaccuracy in the context of their health care, and thus would avoid exposure to this type of intervention. Therefore, any reporting bias would not likely effect the outcome evaluation."

21) CONSORT: Generalisability (external validity, applicability) of the trial findings

21-i) Generalizability to other populations

"A population-based approach was used in conducting this study, identifying all potential smokers within several different survivorship programs in the US and Canada, which contributes to the external validity of the findings. Data were conservatively analyzed using intention-to-treat. This study builds on the previous, effective PFH intervention, and was designed to determine how best to deliver that intervention in a more disseminable format. The study design emphasized external validity and maximizing generalizability of study findings."

21-ii) Discuss if there were elements in the RCT that would be different in a routine application setting

N/A

22) CONSORT: Interpretation consistent with results, balancing benefits and harms, and considering other relevant evidence

22-i) Restate study questions and summarize the answers suggested by the data, starting with primary outcomes and process outcomes (use)

"This paper assesses the comparative efficacy of a print vs. web version of the Partnership for Health-2 intervention. Both interventions were developed to address scalability of smoking cessation interventions among childhood and young adult cancer survivors. PFH-2 was designed to translate core components of PFH (Emmons et al. 2005) to more disseminable formats, and to determine whether equivalent levels of cessation could be achieved.   Equivalent rates of cessation were found in the two arms, and there were no differences in quit rates or changes in readiness to quit. Both interventions were viewed as substantive and appealing and were relatively comparable in terms of intervention “dose” based on participant report of use. These findings suggest that either the print or web-format intervention could be recommended for survivors who smoke, as these cessation interventions yield equivalent levels of success to those found with a telephone-delivered intervention."

22-ii) Highlight unanswered new questions, suggest future research

"Future studies might investigate the inclusion of additional intervention strategies for those in earlier stages of change, such as motivational interviewing as a prelude to access to a web- or print-based intervention."

"Our findings suggest that while access is important, alone it may not be sufficient in this high-risk population to achieve large-scale increases in pharmacotherapy use. This is an area that warrants further research attention."

"This study highlights the need to develop an effective infrastructure for delivery of smoking cessation services to childhood and young adult cancer survivors. The infrastructure for identifying smokers within long-term survivorship care programs is largely missing (de Moor et al. 2007) and a more systematic approach to patient tracking and follow-up is needed."

Other information

23) CONSORT: Registration number and name of trial registry

Clinical Trials Registration No: NCT00588107

24) CONSORT: Where the full trial protocol can be accessed, if available

The study protocol is available upon request to the first author.

25) CONSORT: Sources of funding and other support (such as supply of drugs), role of funders

"This study was supported by grants from the National Cancer Institute, (5R01CA106914-5 and 1K05 CA124415)."

X26-i) Comment on ethics committee approval

"PFH-2 was conducted in collaboration with five cancer centers in the United States and Canada, with Institutional Review Board (IRB) approval at all sites."

x26-ii) Outline informed consent procedures

"A preliminary screen for eligiblity was performed at each site via medical record review or brief telephone screening. After consent for sharing contact information was obtained, contact information was forwarded to the survey team to verify eligibility, obtain informed consent for study participation, and administer the baseline telephone survey."

X26-iii) Safety and security procedures

The survey staff were trained to refer participants, in the unlikely event of distress, to the study investigator, a licensed psychologist.

X27-i) State the relation of the study team towards the system being evaluated

The investigators did not have any conflicts of interest to declare.
